# Effects of white matter hyperintensity on cognitive function in PD patients: a meta-analysis

**DOI:** 10.3389/fneur.2023.1203311

**Published:** 2023-08-09

**Authors:** Wenhao Zhao, Bo Cheng, Tao Zhu, Yingjuan Cui, Yao Shen, Xudong Fu, Maogeng Li, Yuliang Feng, Shushan Zhang

**Affiliations:** ^1^Department of Neurology, Affiliated Hospital of Medical College, North Sichuan Medical College, Nanchong, China; ^2^Department of Preventive Medicine, North Sichuan Medical College, Nanchong, China; ^3^Department of Nursing, Affiliated Hospital of Medical College, North Sichuan Medical College, Nanchong, China

**Keywords:** Parkinson’s disease, white matter hyperintensity, cognitive impairment, dementia, meta-analysis

## Abstract

**Background:**

Parkinson’s disease (PD) is often accompanied by cognitive dysfunction, which imposes a heavy burden on patients, their families, and society. Early identification and intervention are particularly important, but reliable biomarkers for identifying PD-related cognitive impairment at an early stage are currently lacking. Although numerous clinical studies have investigated the association between brain white matter hyperintensity (WMH) and cognitive decline, the findings regarding the relationships between WMH and cognitive dysfunction in PD patients have been inconsistent. Therefore, this study aims to conduct a meta-analysis of the effect of WMH on PD cognitive function.

**Methods:**

This study was conducted in accordance with the Preferred Reporting Items for Systematic Reviews and Meta-Analyses (PRISMA) and Meta-Analysis of Observational Studies in Epidemiology (MOOSE) guidelines. We systematically searched relevant literature from databases such as PubMed, Web of Science, EMBASE, CNKI, and CBM. The retrieval time was limited to database records created up until December 31, 2022. Additionally, we manually retrieved references for full-text reading. Statistical data analysis was performed using RevMan 5.3 and Stata 15.0 software.

**Results:**

This study encompassed 23 individual studies and involved 2,429 patients with PD. The group of PD with mild cognitive impairment (PD-MCI) exhibited a significantly higher overall level of WMH than the group of PD with normal cognitive function (PD-NC) (SMD = 0.37, 95% CI: 0.21–0.52, *p* < 0.01). This finding was consistent across subgroup analyses based on different ethnicities (Asian or Caucasian), WMH assessment methods (visual rating scale or volumetry), and age matching. In addition to the overall differences in WMH load between the PD-MCI and PD-NC groups, the study found that specific brain regions, including periventricular white matter hyperintensity (PVH) and deep white matter hyperintensity (DWMH), had significantly higher WMH load in the PD-MCI group compared to the PD-NC group. The study also conducted a meta-analysis of WMH load data for PD with dementia (PDD) and PD without dementia (PDND), revealing that the overall WMH load in the PDD group was significantly higher than that in the PDND group (SMD = 0.98, 95% CI: 0.56–1.41, *p* < 0.01). This finding was consistent across subgroup analyses based on different ethnicities and age matching. Moreover, regarding specific brain regions (PVH or DWMH), the study found that the PDD group had significantly higher WMH load than the PDND group (*p* < 0.01).

**Conclusion:**

WMH was associated with PD cognitive dysfunction. The early appearance of WMH may indicate PD with MCI.

## Introduction

Parkinson’s disease (PD) is the second most prevalent neurodegenerative disorder after Alzheimer’s disease (AD). With the aging of the population, its incidence and prevalence have been increasing in recent years. Epidemiological studies have shown that PD affects 0.3% of the general population in developed countries, 1.0% of people aged over 60 years, and 3.0% of those over 80 years old (1). Initially, PD was characterized as a movement disorder with core motor symptoms such as resting tremors, bradykinesia, postural instability, and stiffness in the neck, trunk, and limbs. However, it is now recognized that PD also presents with non-motor symptoms (NMS) such as olfactory dysfunction, constipation, autonomic dysfunction, sleep disorders, cognitive impairment, anxiety, and depression (2). Clinical studies indicate that NMS may manifest several years or even decades before the onset of motor symptoms, which may have important diagnostic implications (3).

Cognitive dysfunction is a NMS in PD, encompassing both PD with mild cognitive impairment (PD-MCI) and PD with dementia (PDD). Clinical studies indicate that approximately 30% of newly diagnosed PD patients develop PD-MCI (4), while PDD affects roughly 80% of PD patients who have had the disease for over 10 years (5). Previous research has identified various risk factors for cognitive dysfunction in PD, including male gender, advanced age, higher Hoehn and Yahr scale stage, severity of motor symptoms, speech impairment, postural instability/gait difficulty subtype, depression, hallucinations, and educational level (6).

PD-MCI is a clinical syndrome characterized by cognitive and functional deficits that exhibit heterogeneity. It represents an intermediate stage between normal cognition and dementia, and can involve one or more cognitive domains. Clinical studies indicate that PD-MCI is an early stage of cognitive decline in PD and a significant risk factor for the progression of PD to PDD (7).

As PDD typically develops within an already established diagnosis of PD, detecting and diagnosing PD before the onset of dementia symptoms is crucial. PDD has a subtle onset and slow progression, primarily affecting attention, executive function, visual–spatial abilities, memory, and other cognitive domains. Executive dysfunction is particularly prominent and often accompanied by hallucinations, delusions, apathy, and emotional or personality changes (8). Compared with AD patients, there are some differences in the degree and features of cognitive deficits in individual cognitive domains: memory impairment is more pronounced in AD, while executive dysfunction is more common in PDD. Several risk factors have been identified for PDD, including age, time of diagnosis, akinetic-rigid subtype, disease severity, verbal fluency impairments, genetic factors, low education level, and postural instability (9).

PD-MCI can impact the lives of PD patients by diminishing their ability to communicate, access social support, and perform daily activities, which can be especially daunting for young patients facing societal, familial, and occupational pressures. In comparison, PDD has an even greater impact on the lives of PD patients (10). Hence, early and precise identification and diagnosis of cognitive dysfunction in PD are critical for reducing harm and enhancing patient outcomes. However, there is currently a lack of biological markers to accurately detect cognitive dysfunction in PD in clinical practice.

White matter hyperintensity (WMH), also known as leukoaraiosis (LA), is typically observed as merged or patchy areas of high-signal intensity on T2-weighted imaging (T2 WI) or fluid-attenuated inversion recovery (FLAIR) sequences in magnetic resonance imaging (MRI) scans of older adults (11). Generally, WMH is considered an imaging marker of white matter damage that increases with age, and the detection rate of WMH in Asians is generally significantly higher than that in Caucasians (12). In neuroimaging, WMH is typically classified into two subtypes: periventricular white matter hyperintensity (PVH) and deep white matter hyperintensity (DWMH). Research has found that WMH reflects chronic hypoperfusion of the brain’s white matter, indicating axonal injury, myelin sheath damage, and gliosis. The core pathophysiological mechanism involves vascular damage caused by ischemia and hypoxia (13–16). Related studies have also found that WMH is involved in the entire process of cognitive impairment (17–22). In an 8-year cohort study, Kuller et al. found that individuals with significant WMH had a significantly increased risk of developing AD (HR = 1.5, 95%CI: 1.17–1.99) (23). Silbert et al. found that compared with baseline WMH load, WMH progression may be the most crucial risk factor for predicting cognitive dysfunction (19). Clinical studies have also found that WMH in different areas may have varying effects on cognitive function. Smith et al. found that executive dysfunction and memory were associated with the specific location of WMH rather than the overall volume of WMH (24). Subsequently, Sunwoo et al. found that WMH load (OR: 1.616, *P* < 0.01) and Cholinergic Pathways Hyperintensities Scale (CHIPS) score (OR: 1.084, *P* < 0.01) were related to the outcome of PDD (25). Lee et al. found that WMH was an independent factor related to PD cognitive dysfunction, regardless of age, gender, disease duration, severity, and cerebrovascular risk factors (26). However, Lee et al. (27) found that baseline WMH load was not related to dementia but longitudinal follow-up showed that WMH could predict the occurrence of PDD. In a cohort study, Hanning et al. (28) found that total WMH load was not related to the cognitive function of newly diagnosed PD patients. Overall, previous literature reports indicate significant differences in the relationship between WMH and PD cognitive impairment.

In recent years, there has been an increasing number of studies exploring the impact of WMH on cognitive impairment in PD. Therefore, this study aims to conduct a systematic review and meta-analysis of literature published domestically and internationally in recent years regarding the effect of WMH on cognitive impairment in PD. The objective is to provide further clarification on the impact of WMH on cognitive function in PD, explore imaging markers for early identification of cognitive dysfunction in PD, and offer new evidence for early recognition and intervention of cognitive dysfunction in clinical practice.

## Methods

### Study design

This study adheres to the Meta-analysis of Observational Studies in Epidemiology (MOOSE) guidelines for epidemiological observational studies and follows the Preferred Reporting Items for Systematic Reviews and Meta-Analyses (PRISMA) recommendations for conducting systematic reviews and meta-analyses.

### Study search and selection

This study systematically searched relevant literature in online databases, including PubMed, Web of Science, EMBASE, China National Knowledge Infrastructure (CNKI), and Chinese Biomedical Literature Database (CBM). The search was limited to the period from each database’s establishment to December 31, 2022. Additionally, manual searches were conducted on the reference lists of full-text articles. Search terms used in this study mainly originated from subject headings and free words. The following search terms were utilized:

“Parkinson Disease,” “Parkinson’s Disease,” “Parkinsonism,” “paralysis agitans,” “white matter hyperintensities,” “white matter hyperintensity,” “white matter lesion,” “white matter lesions,” “small vessel disease,” “leukoaraiosis,” “leukoencephalopathy,” “leukoencephalopathies,” “cognitive dysfunction,” “cognitive dysfunctions,” “cognitive decline,” “cognitive declines,” “cognitive impairment,” “cognitive impairments,” “neurocognitive disorder,” “neurocognitive disorders,” “mental deterioration,” and “dementia.”

The included studies met the following criteria: (1) PD patients included in this study were diagnosed according to the United Kingdom Parkinson’s Disease Society Brain Bank Criteria from 1992 and/or the clinical diagnostic criteria for PD established by the Movement Disorders and Parkinson’s Disease Group of the Chinese Medical Association Neurology Branch in 2016 and/or the Parkinson’s disease diagnostic criteria by the Movement Disorder Society (MDS) in 2015. (2) The study provided relevant data on the cognitive function status grouping of PD patients (cognitive impairment group and non-cognitive impairment group) and the identification and quantification of WMH severity based on head MRI. (3) The study type was limited to cohort studies or case–control studies. (4) Studies that could provide mean ± standard deviation data suitable for meta-analysis were included. (5) The publication language was limited to Chinese or English.

The exclusion criteria were as follows: (1) PD patients with a history of or coexisting central nervous system diseases, peripheral nerve diseases that affect movement and/or autonomic function, and psychiatric illnesses unrelated to Parkinson’s disease were excluded from this study. (2) Animal experiments were also excluded from this study. (3) Literature such as case reports, reviews, and commentaries that could not provide mean ± standard deviation data suitable for this study were excluded.

Investigators WZ and BC independently screened titles and abstracts of articles and determined whether to search for further articles based on the inclusion and exclusion criteria. Articles that could not be excluded were retrieved, and their full text was reviewed by TZ and YC. For articles with insufficient reported data for analysis, we contacted the corresponding author *via* email to request additional data. Any disagreements were resolved through discussions between the reviewers, and third reviewer SZ was consulted when necessary.

### Data extraction

Two reviewers independently extracted relevant data, including the first author, publication year, research location, sample size, race, age, gender composition ratio, PD disease duration, Hoehn-Yahr stage, revised Unified Parkinson’s Disease Rating Scale part III (UPDRS III), Montreal Cognitive Assessment (MoCA) or Mini-Mental State Examination (MMSE) scores, WMH distribution location, WMH evaluation method, and mean ± standard deviation data used for meta-analysis.

### Quality assessment

The quality of the included literature was evaluated using the Newcastle-Ottawa Scale (NOS) (29), which consists of eight items and uses a semi-quantitative star system to assess the quality of the literature, with a maximum score of nine stars. The items include selection of study groups, comparability, and exposure or outcome evaluation. Studies with an NOS score of 5 or higher are considered to be of high quality (30). Additionally, Egger’s tests were performed using Stata version 15.0 to quantitatively analyze potential publication bias.

### Data synthesis and statistical analysis

RevMan 5.3 software was used for statistical analysis in this study. Continuous variables were represented using the weighted mean difference (MD) or standardized mean difference (SMD) and their corresponding 95% confidence intervals (CI). If the same method was used to assess WMH and cognitive function across studies, MD and its corresponding 95% CI were used for result analysis; otherwise, SMD and its corresponding 95% CI were used. The combined MD or SMD and their 95% CIs were calculated using either a random effects model or fixed effects model, and forest plots were generated to present individual studies and summary data. Heterogeneity between studies was assessed using the chi-square test and the I^2^ statistic, with significant heterogeneity being considered when *p* < 0.1 for the chi-square test or *I*^2^ > 50% (31–33). A random effects model was used for statistical analysis if significant heterogeneity was present; otherwise, a fixed effects model was employed (31–33). Sensitivity analyses were performed by omitting one study at a time to evaluate the robustness of the results.

## Results

### Search results

**Figure 1:**
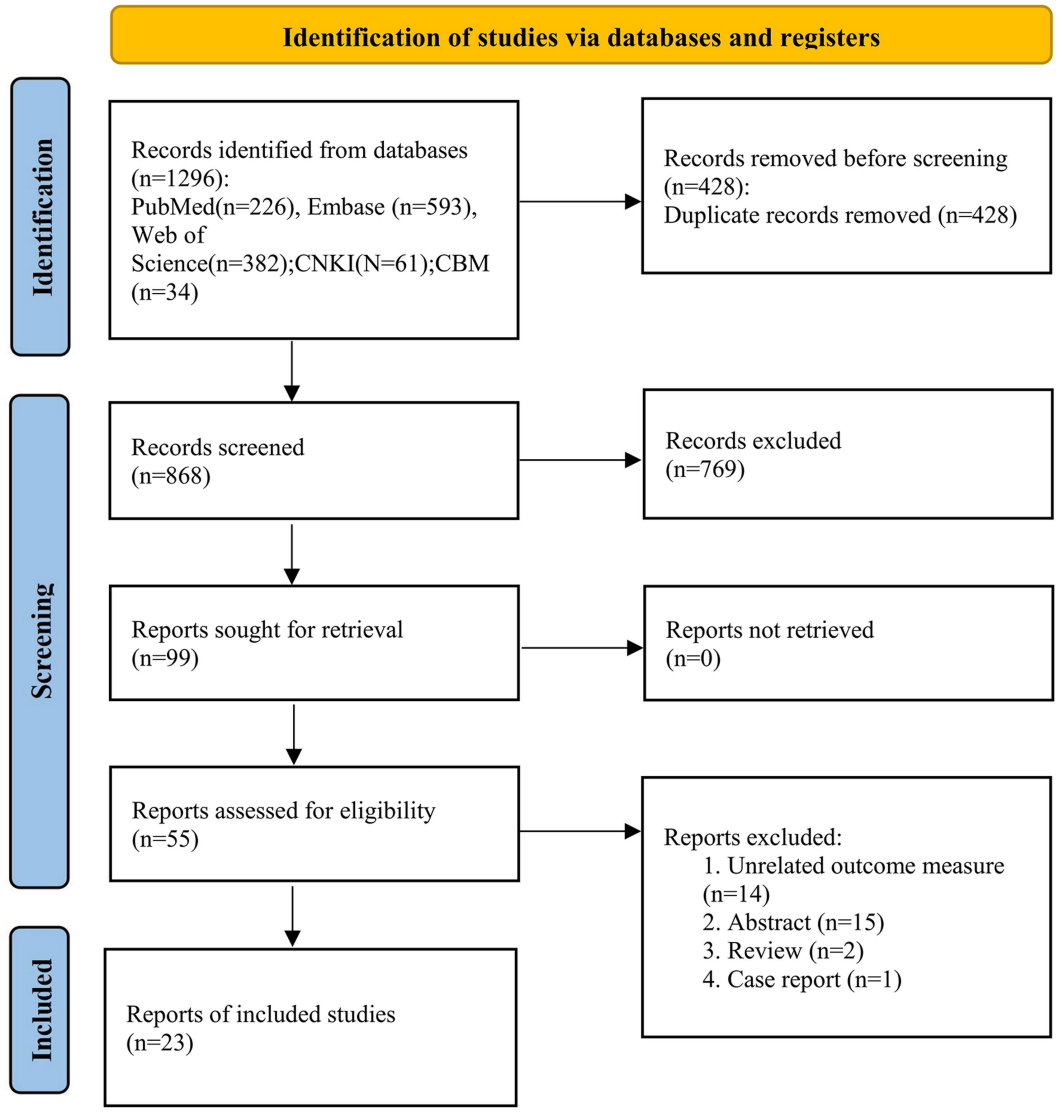
PRISMA search strategy flow diagram of studies selection process.

Using the established retrieval strategy and selected online databases, a total of 1,296 literature records were obtained. Through manual deduplication combined with literature management software, we reviewed and organized the titles and abstracts of 868 literature records and retrieved and read the full text of 99 articles. Finally, 23 studies were included in the meta-analysis. The literature search process is shown in Figure 1.

### Study characteristics

This study included a total of 23 relevant studies involving 2,429 patients with PD. The publication dates of the included studies ranged from February 2006 to February 2021. Among them, 17 studies compared the severity of WMH between PD-MCI and PD-NC, while 7 studies compared the WMH status between PDD and PD without dementia (PDND). Only one study (34) simultaneously compared the WMH status between PD-MCI and PD-NC groups, as well as between PDD and PDND groups. The largest study enrolled 192 PD patients, while the smallest study enrolled 28. Of the included studies, 14 were from Asia, 8 were from Europe, and 1 was from the Americas. Except for two studies (35, 36), all included studies provided information on the course of PD. The evaluation of WMH was mainly performed through visual assessment or volumetric analysis of MRI scans of PD patients, with corresponding scale assessment data provided. Among them, 15 studies used visual assessment, 7 studies used volumetric analysis, and only one study (37) used both visual and volumetric assessment. The grouping of PD patients based on the degree of cognitive impairment was mainly determined by MMSE scores and/or MoCA scale. However, three studies did not use MMSE or MoCA to evaluate the degree of cognitive impairment in PD patients. One study (34) assessed the degree of cognitive impairment using various tests such as the Rey Auditory Verbal Learning Test (RAVLT), the Verbal Fluency Test (VFT), the Stroop Color-Word Test (SCWT), the Trail Making Test Part B (TMT-B), the Raven’s Colored Progressive Matrices (RCPM), the Clock Drawing Test, and the Token Test. Another study (38) utilized the Korean version of MMSE, the Clinical Dementia Rating (CDR) scale, and the Clinical Dementia Rating sum of boxes (CDR-SB) to evaluate the degree of cognitive impairment in PD patients. Finally, one study (37) employed MMSE scores and the Mattis Dementia Rating Scale to evaluate the severity of cognitive impairment, but no MMSE-related data were found in the latter two studies. All of the included studies were assessed as high-quality studies according to the NOS. The basic characteristics of the included studies and the results of quality assessment are shown in Tables 1–3.

**Table 1:** Basic characteristics and quality assessment of the included studies in PD-NC and PD-MCI groups.

First author/time	Age (years) N/M	Sample size N/M	Male (%) N/M	Country/Ethnicity	Duration of PD (years) N/M	UPDRS-III N/M	Location of WMH	WMH assessment	H-Y N/M	NOS
Dalaker et al. (39)	65.5 ± 9.2/ 69.4 ± 7.8	133/30	60.2%/63.3%	America/Caucasian	27.8 ± 19.7^a^/26.4 ± 20.8^a^	21.4 ± 9.9/23.7 ± 11.1	T	Volumetric	1.8 ± 0.6/2.0 ± 0.5	8
Kim et al. (38)	63.4 ± 12.0/70.0 ± 6.8	25/48	56.0%/33.3%	Korea/Asian	1.8 ± 0.8/1.9 ± 0.8	NA	T	Visual	1.4 ± 0.5/1.7 ± 0.8	5
Shin et al. (40)	66.6 ± 6.4/69.5 ± 6.9	44/87	40.9%/39.1%	Korea/Asian	2.2 ± 2.03/3.1 ± 3.06	17.9 ± 9.9/19.0 ± 9.3	T	Visual	NA	8
Kandiah et al. (41)	63.3 ± 7.5/68.9 ± 6.1	67/24	70.15%/75.00%	Singapore/Asian	5.3 ± 4.2/4.9 ± 2.6	NA	T/P/D	Volumetric	1.9 ± 0.3/1.8 ± 0.8	8
Wang et al. (42)	63.0 ± 8.0/65.0 ± 8.0	23/23	65.2%/47.80%	China/Asian	2.3 ± 1.7/3.1 ± 2.6	26 ± 12/32 ± 13	T/F/Pa/O/Te/D/B/I	Visual	1.9 ± 0.6/2.1 ± 0.7	7
Ham et al. (35)	69.0 ± 6.1/70.3 ± 8.1	41/46	36.6%/54.30%	Korea/Asian	NA	22.9 ± 9.4/29.4 ± 10.9	T	Visual	NA	8
Amboni et al. (43)	65.8 ± 6.5/65.2 ± 8.7	21/21	66.7%/85.70%	Italy/Caucasian	5.9 ± 2.6/6.6 ± 3.7	13.1 ± 5.3/14.3 ± 8.5	T	Volumetric	1.5 ± 0.6/1.5 ± 0.4	8
Baggio et al. (44)	64.0 ± 9.8/66.1 ± 12.2	43/22	53.5%/63.60%	Spain/Caucasian	10.8 ± 5.1/8.8 ± 4.0	14.1 ± 7.5/18.2 ± 8.7	T	Volumetric	NA	8
Mak et al. (45)	63.4 ± 7.6/ 69.4 ± 6.4	65/25	72.3%/76.00%	Singapore/Asian	5.4 ± 4.3/5.0 ± 2.7	17.5 ± 7.0/20.0 ± 8.4	T/P/D	Volumetric	1.9 ± 0.4/1.8 ± 0.4	8

^a^This item is measured in months.

**Table 2:** Basic features and quality evaluation of included studies in PD-NC and PD-MCI groups.

First author/time	Age (years) N/M	Sample size N/M	Male (%) N/M	Country/Ethnicity	Duration of PD (years) N/M	UPDRS-III N/M	Location of WMH	WMH assessment	H-Y N/M	NOS
Oh et al. (46)	62.5 ± 9.4/68.8 ± 6.6	53/76	50.9%/42.10%	Korea/Asian	1.9 ± 1.8/ 1.7 ± 1.6	NA	T	Visual	1.5 ± 0.7/1.7 ± 0.6	7
Li et al. (47)	63.45 ± 7.65/64.28 ± 8.05	45/43	53.33%/51.16	China/Asian	2.85 ± 1.38/ 3.46 ± 2.25	25.85 ± 10.57/33.65 ± 12.46	P/D/B/I	Visual	1.80 ± 0.50/2.27 ± 0.65	8
Dunet et al. (37)	78.1 ± 6.1/81.8 ± 3.8	15/13	67%/54%	Switzerland, France/Caucasian	5.7 ± 3.6/ 6.5 ± 5.1	NA	T	Visual/Volumetric	NA	6
Stojkovic et al. (48)	61.5 ± 8.1/65.6 ± 7.9	46/61	65.2%/64%	Italy/Caucasian	7.2 ± 5.4/ 8.9 ± 5.3	36.5 ± 13.6/44.5 ± 11.9	T	Volumetric	2.1 ± 0.9/2.4 ± 0.8	8
Hanning et al. (28)	64.0 ± 9.0 63.0 ± 10.0	29/79	59%/69%	Germany/Caucasian	NA	14 (8; 22)/18 (14;28)		Visual	1.5 (1;2)/2 (1.3;2.5)	6
Huang et al. (49)	61.3 ± 9.5/65.4 ± 7.8	81/94	58.0%/55.3%	Singapore/Asian	14.85 ± 9.05^a^/10.97 ± 7.29^a^	17.3 ± 7.5/25.1 ± 11.1	T/P	Volumetric	1.7 ± 0.4/1.8 ± 0.4	8
Li et al. (50)	60.4 ± 3.3/60.2 ± 3.0	30/29	56.7%/58.6%	China/Asian	2.5 ± 1.0/5.7 ± 1.1	10.3 ± 1.9/21.6 ± 2.5	P/D /T/O/Te/F/B/I	Visual	1.3 ± 0.3/2.2 ± 0.2	7
Nicoletti et al. (34)	64.4 ± 10.4/67.5 ± 7.4	84/55	61.9%/63.6%	Italy/Caucasian	3.0 ± 2.9/3.0 ± 2.7	25.4 ± 14.5/27.4 ± 11.9	T	Visual	1.9 ± 0.6/2.2 ± 0.7	8

NA, not available; UPDRS-III, Unified Parkinson’s Disease Rating Scale Part III; MMSE, Mini-Mental State Examination; MoCA, Montreal Cognitive Assessment; H-Y staging, Hoehn and Yahr staging; NOS, Newcastle-Ottawa Scale; N, PD-NC; M, PD-MCI; T, Total WMH (White matter hyperintensity); D, Deep; P, Periventricular; I, Infratentorial; B, Basal ganglia; O, Occipital lobe; Te, Temporal lobe; Pa, Parietal lobe; F, Frontal lobe; The data is presented as mean ± standard deviation or percentage; a, This item is measured in months; b, Data is presented as median (first quartile; third quartile).

**Table 3:** Basic characteristics and quality assessment of the included studies in PDND and PDD groups.

First author/time	Age (years) N/D	Sample size N/D	Male (%) N/D	Country/Ethnicity	Duration of PD (years) N/D	UPDRS-III N/D	Location of WMH	WMH Assessment	H-Y N/D	NOS
Beyer et al. (51)	71.3/ 73.9	19/16	52.6%/ 62.50%	Norway/Caucasian	12.7 ± 6.4/12.5 ± 7.4	27.2 ± 13.1/42.3 ± 12.4	T, P, F、Pa、O、DW、B、I	Visual	2.3 ± 0.6/3.1 ± 0.6	8
Daida et al. (52)	62.2 ± 10.3/ 70.7 ± 9.0	103/21	38.8%/ 81.9%	Japan/Asian	11.1 ± 6.2/9.5 ± 6.3	NA	T、P、DW	Visual	2.8 ± 0.8/3.6 ± 0.9	8
Lee et al. (26)	65.5 ± 6.5/ 70.2 ± 7.0	11/35	54.5%/ 31.40%	Korea/ Asian	1.4 ± 1.06/1.9 ± 1.7	8.5 ± 7.1/22.5 ± 12.7	T、P、DW、I、B、	Visual	1.5 ± 0.7/2.2 ± 0.9	7
Joki et al. (36)	74.5 ± 5.5/ 75.7 ± 6.2	50/50	52%/ 64%	Japan/Asian	NA	NA	T、P、DW	Visual	NA	8
Sławek et al. (53)	61.9 ± 9.1/ 67.9 ± 8.7	135/57	45.9%/ 50.9%	Poland/Caucasian	5.9 ± 4.6/8.7 ± 6.3	29.6 ± 15.3/ 42.9 ± 18.9	T、P、DW	Visual	2.0 ± 0.7/2.4 ± 0.7	8
Nicoletti et al. (34)	65.3 ± 9.5/	121/18	66.7% /66.7%	Italy/Caucasian	2.9 ± 2.8/3.4 ± 2.8	25.1 ± 12.7/33.1 ± 16.5	T	Visual	2.1 ± 0.7/1.9 ± 0.6	8
Tanaka et al. (54)	62.2 ± 10.2/71.6 ± 8.4	27/110	40.0%/ 70.40%	Japan/Asian	11.1 ± 6.1/10.1 ± 6.7	NA	P、DW	Visual	2.8 ± 0.8/3.7 ± 0.8	6

NA, not available; UPDRS-III, Unified Parkinson’s Disease Rating Scale part III; MMSE, Mini-Mental State Examination; MoCA, Montreal Cognitive Assessment; HY, Hoehn and Yahr scale stage; NOS, Newcastle-Ottawa Scale; N, PDND; D, PDD; T, total WMH; DW, deep matter; P, periventricular matter; I, infratentorial matter; B, basal ganglia; O, occipital lobe; Te, temporal lobe; Pa, parietal lobe; F, frontal lobe. The data is presented as mean ± standard deviation or percentage.

### Quality assessment

The NOS scores of the included studies ranged from 5 to 8, with no study being assessed as low quality. The methodological quality of the included meta-analysis studies is detailed in Tables 1–3. The Egger test using Stata 15 showed no evidence of publication bias.

### Effects of total WMH in PD-MCI versus PD-NC

**Figure 2:**
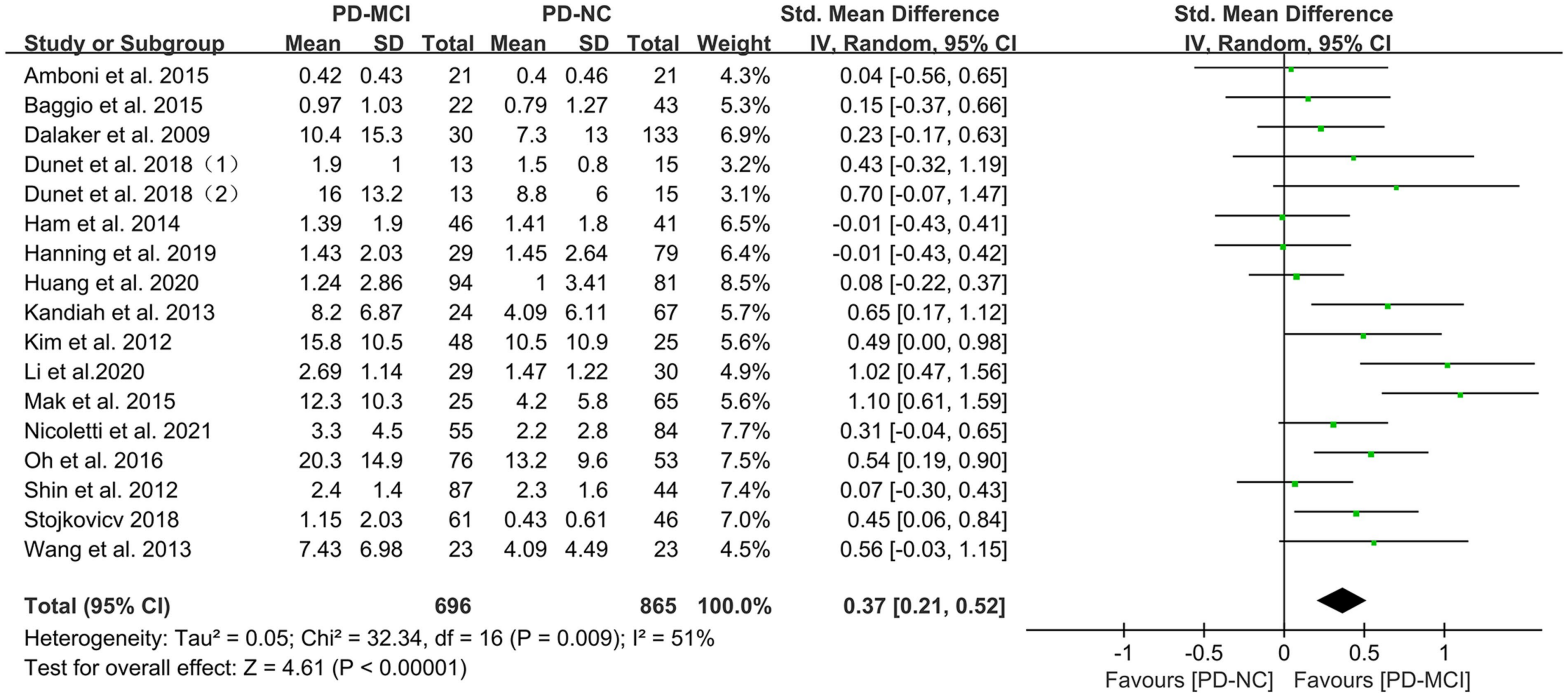
Forest plot and meta-analysis of total WMH between PD-MCI and PD-NC.

Data comparing the total WMH load between PD-MCI and PD-NC groups were available in 17 studies (28, 34, 35, 37–46, 48–50), which included a total of 1656 PD patients. Due to high heterogeneity (32), a random-effects model was used for statistical analysis. The meta-analysis results showed that the overall WMH load in the PD-MCI group was significantly higher than that in the PD-NC group (SMD = 0.37, 95% CI: 0.21–0.52, *p* < 0.01), as presented in Figure 2.

**Figure 3:**
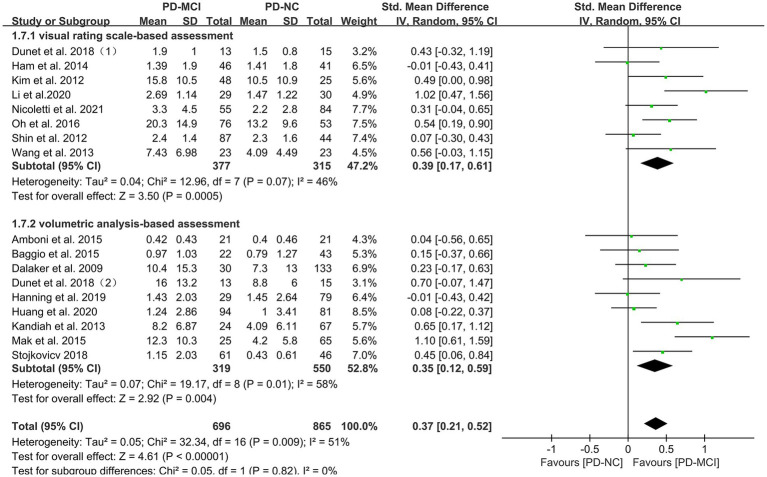
Forest plot and meta-analysis of WMH between PD-MCI and PD-NC: a subgroup analysis based on different assessment modalities.

**Figure 4:**
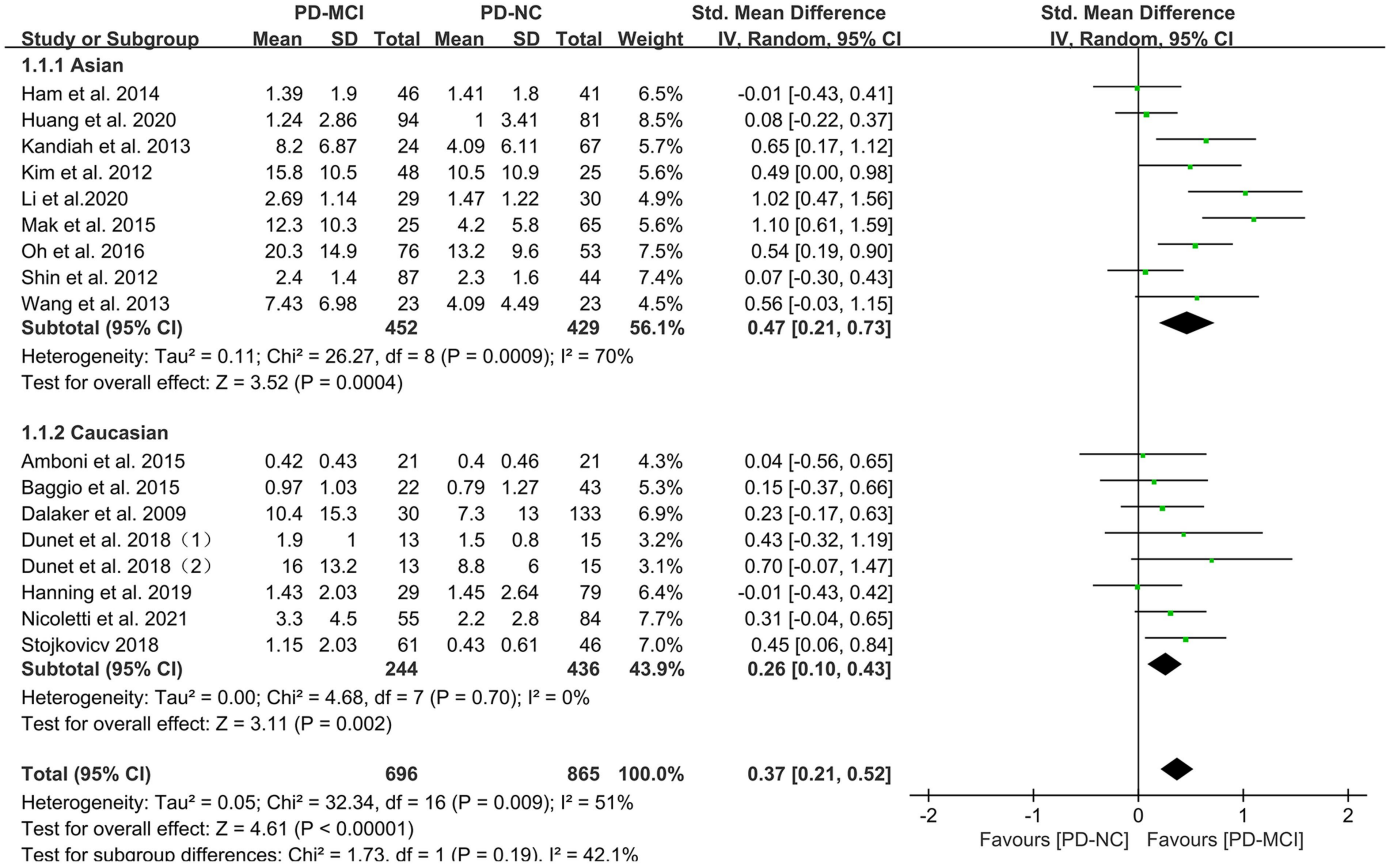
Forest plot and meta-analysis of WMH between PD-MCI and PD-NC: a subgroup analysis based on different ethnicities.

**Figure 5:**
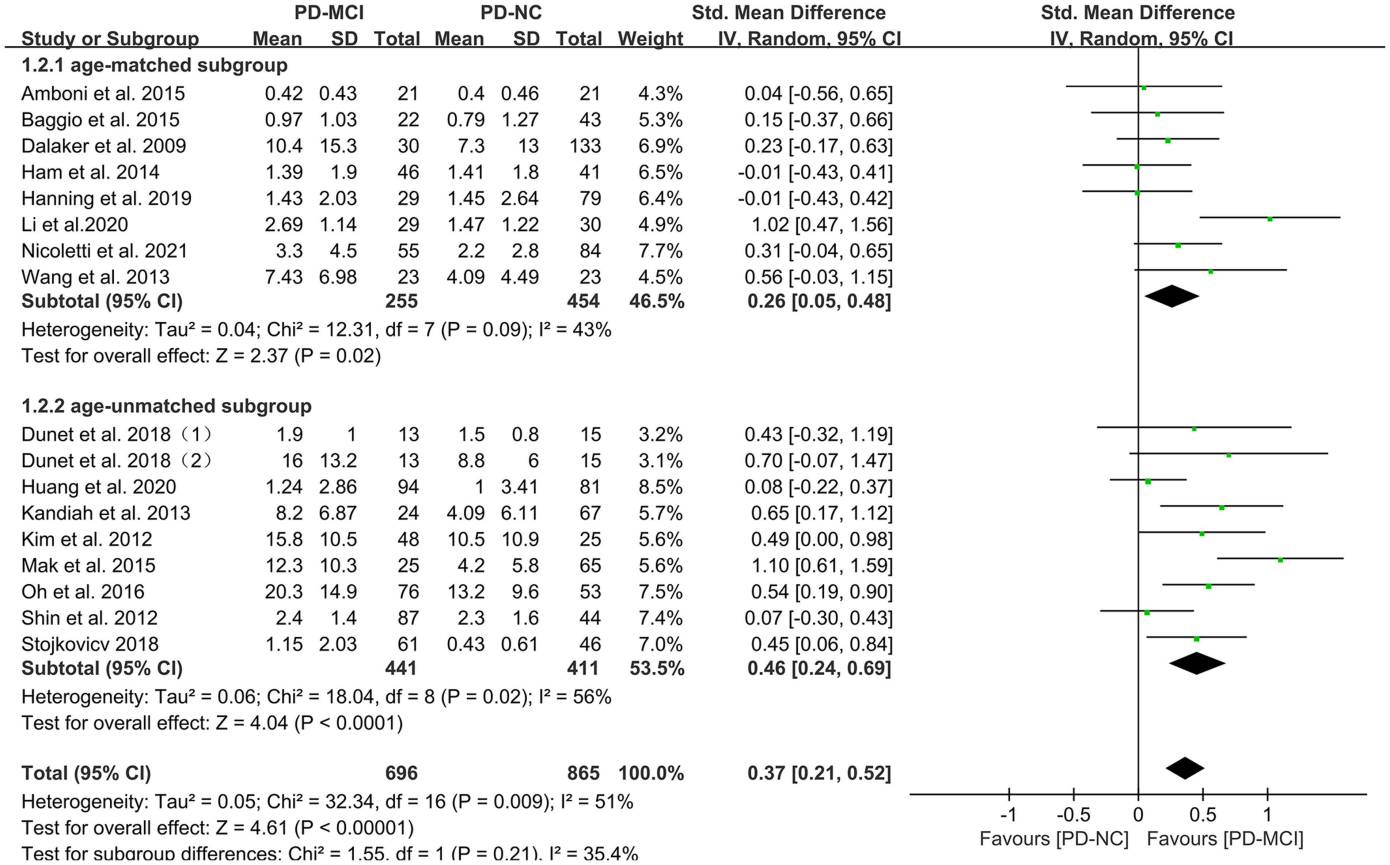
Forest plot and meta-analysis of WMH between PD-MCI and PD-NC: a subgroup analysis based on age.

Subgroup analyses were performed based on relevant factors of interest in clinical practice, such as different WMH assessment methods (visual rating scale and volumetric analysis), PD patients’ belonging to different ethnic groups (Asian or Caucasian) and whether they were age-matched. The subgroup analyses revealed that: (1) both visual rating scale-based assessment (SMD = 0.39, 95% CI: 0.17–0.61, *p* < 0.01) and volumetric analysis-based assessment (SMD = 0.35, 95% CI: 0.12–0.59, *p* < 0.01) showed significant differences in WMH load between PD-MCI and PD-NC groups, as presented in Figure 3; (2) the WMH load in both Asian (SMD = 0.47, 95% CI: 0.21–0.73, *p* < 0.01) and Caucasian (SMD = 0.26, 95% CI: 0.10–0.43, *p* < 0.01) PD-MCI groups was significantly higher than that in the PD-NC group, as shown in Figure 4; (3) additionally, in the age-matched subgroup (SMD = 0.26, 95% CI: 0.05–0.48, *p* = 0.02) and the age-unmatched subgroup (SMD = 0.46, 95% CI: 0.24–0.69, *p* < 0.01), the WMH load in the PD-MCI group was significantly higher than that in the PD-NC group, as shown in Figure 5.

### Effects of PVH in PD-MCI versus PD-NC

**Figure 6:**
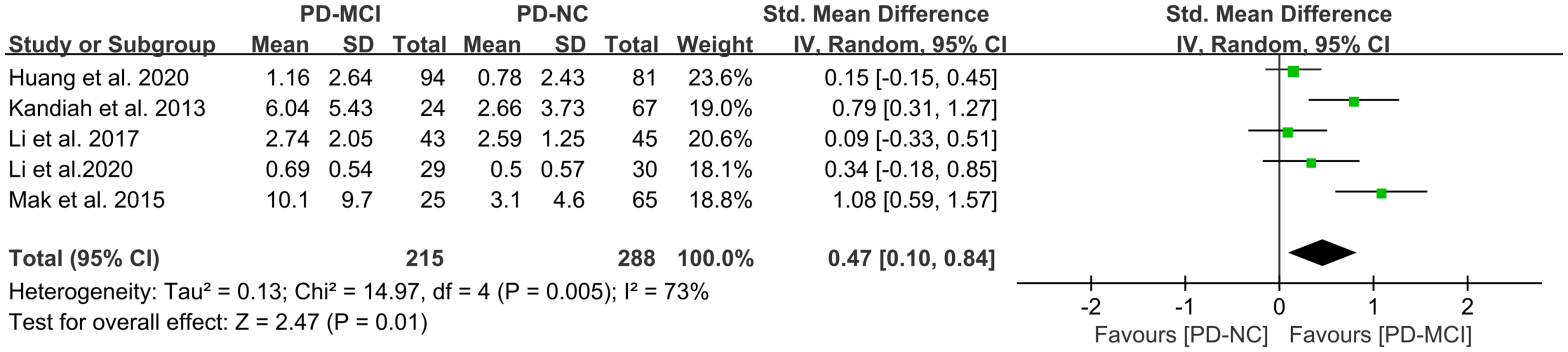
Forest plot and meta-analysis of PVH between PD-MCI and PD-NC.

This study included five studies (41, 45, 47, 49, 50) that compared the PVH load between the PD-MCI and PD-NC groups, with a total of 503 PD patients. The heterogeneity test suggested high heterogeneity; therefore, a random-effects model was used for data analysis. The results indicated that the PVH load in the PD-MCI group was significantly higher than that in the PD-NC group (SMD = 0.47, 95% CI: 0.10–0.84, *p* = 0.01), as demonstrated in Figure 6.

### Effects of DWMH in PD-MCI versus PD-NC

**Figure 7:**
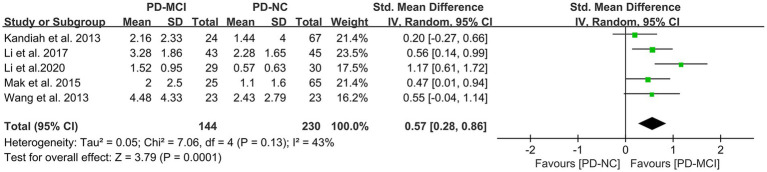
Forest plot and meta-analysis of DWMH between PD-MCI and PD-NC.

A total of five studies (41, 45, 47, 50) met the inclusion criteria and provided relevant data for comparing the DWMH load between the PD-MCI and PD-NC groups, with a total of 374 PD patients. The results of the random-effects model analysis revealed that the DWMH load in the PD-MCI group was significantly higher than that in the PD-NC group (SMD = 0.57, 95% CI: 0.28–0.86, P<0.01), as presented in Figure 7.

### Effects of total WMH in PDD versus PDND

**Figure 8:**
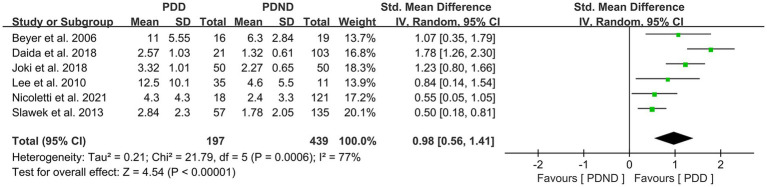
Forest plot and meta-analysis of total WMH between PDD and PDND.

This study included a total of six studies (26, 34, 36, 42, 51–53) that provided data for comparing the total WMH load between the PDD and PDND groups, with a total of 636 PD patients. It is noteworthy that all six studies used visual rating scales to evaluate the severity of WMH. The results of the random-effects model analysis revealed that the overall WMH load in the PDD group was significantly higher than that in the PDND group (SMD = 0.98, 95% CI: 0.56–1.41, *p* < 0.01), as demonstrated in Figure 8.

**Figure 9:**
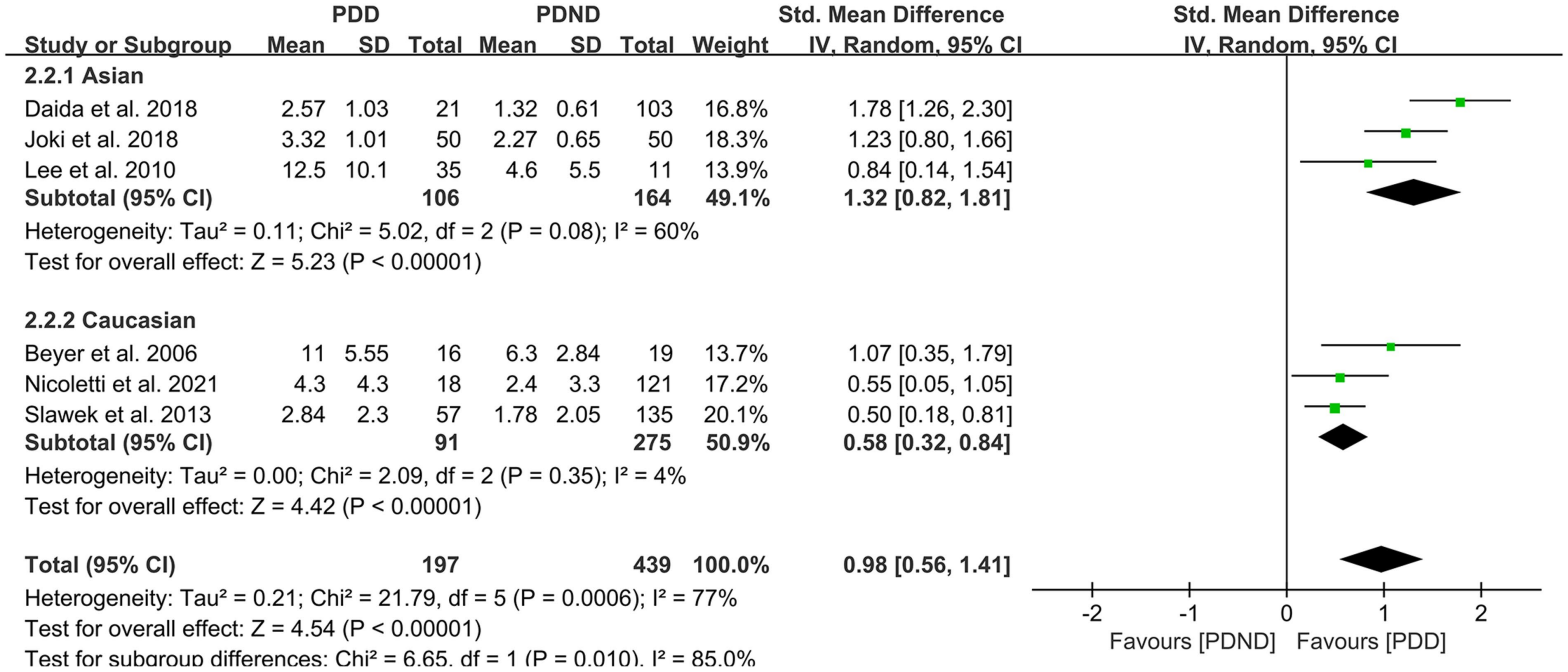
Forest plot and meta-analysis of WMH between PDD and PDND: a subgroup analysis based on different ethnicities.

**Figure 10:**
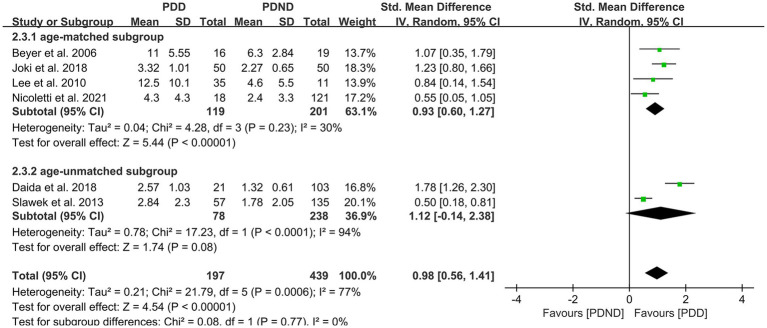
Forest plot and meta-analysis of WMH between PDD and PDND: a subgroup analysis based on age.

Further subgroup analyses based on whether different races and ages were matched found that: (1) among Asians (SMD = 1.32, 95%CI: 0.82–1.81, p < 0.01) and Caucasians (SMD = 0.58, 95% CI: 0.32–0.84, p < 0.01), the overall WMH load in the PDD group was significantly higher than that in the PDND group, as presented in Figure 9; (2) in the age-matched group, the overall WMH load in the PDD group was significantly higher than that in the PDND group (SMD = 0.93, 95% CI: 0.60–1.27, *p* < 0.01); however, there was no significant difference in the total WMH load between the PDD and PDND groups in the non-age-matched group (SMD = 1.12, 95% CI: −0.14-2.38, *p* = 0.08), as shown in Figure 10.

### Effects of PVH in PDD versus PDND

**Figure 11:**
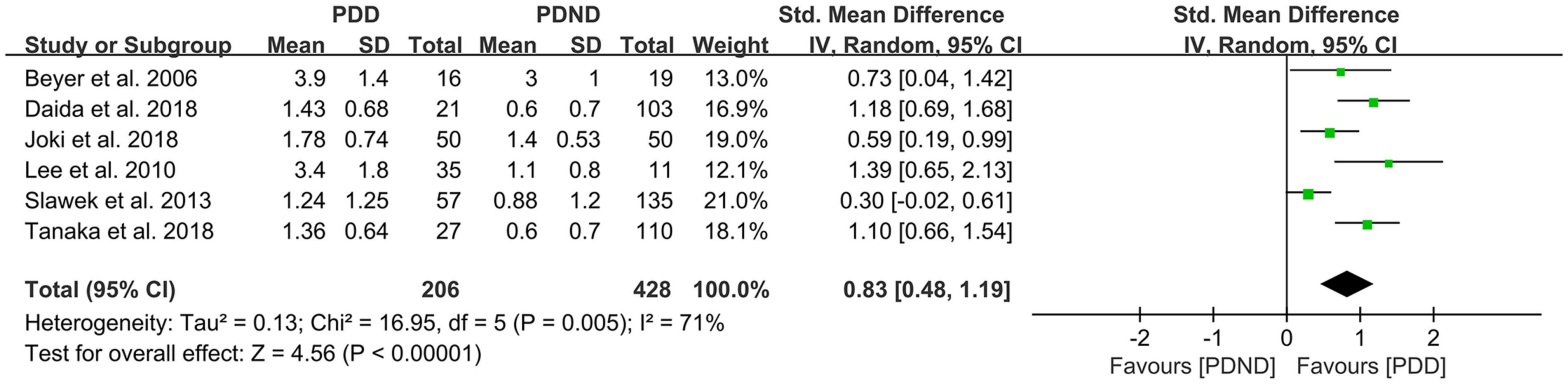
Forest plot and meta-analysis of PVH between PDD and PDND.

This study included a total of six studies (26, 36, 51–54) that provided relevant data for comparing the PVH load between the PDD and PDND groups, with a total of 375 PD patients. The results of the random-effects model analysis revealed that the PVH load in the PDD group was significantly higher than that in the PDND group (SMD = 0.83, 95% CI: 0.48–1.19, *p* < 0.01), as demonstrated in Figure 11.

### Effects of DWMH in PDD versus PDND

**Figure 12:**
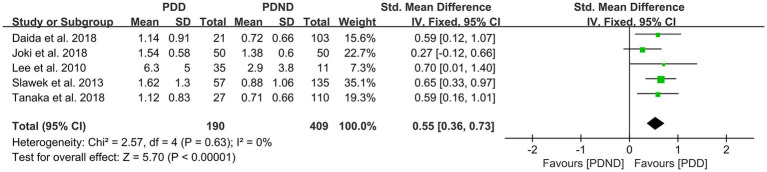
Forest plot and meta-analysis of DWMH between PDD and PDND.

A total of five studies (26, 36, 52–54) reported relevant data for comparing the DWMH load between the PDD and PDND groups. The heterogeneity test showed no significant heterogeneity; therefore, a fixed-effects model was used for statistical analysis. The results revealed no significant difference in DWMH load between the PDD and PDND groups (SMD = 0.55, 95% CI: 0.36–0.73, *p* < 0.01), as presented in Figure 12.

### Sensitivity analysis

This study conducted sensitivity analyses for all explored outcome measures, and the results demonstrated that the study findings were stable and reliable.

## Discussion

Our research systematically retrieved literature on the relationship between WMH and cognitive impairment in PD. We conducted a meta-analysis to investigate the impact of WMH load on cognitive function in PD, which is of clinical significance for doctors. A total of 19 case–control studies and 4 longitudinal cohort studies with 2,429 participants were included. The study found that the severity of WMH may play an important role in the cognitive decline of PD, even in the early stages of cognitive impairment, and may serve as an imaging biomarker for early cognitive dysfunction in PD patients.

In a cross-sectional study, Dalaker et al. (39) compared the total load of WMH in 163 newly diagnosed untreated PD patients and 102 age-matched healthy controls but found no significant difference in the overall WMH load between the two groups. Subgroup analysis of PD-MCI and PD-NC did not reveal any significant differences between the two groups. Similarly, Baggio et al. (44) and Amboin et al. (43) also did not find a relationship between WMH and cognitive impairment in PD. However, Kandiah et al. (41) found that the overall WMH load in PD-MCI patients was significantly higher than in PD-NC patients; even after adjusting for age and vascular risk factors such as diabetes, hypertension, hyperlipidemia, smoking, etc., the overall WMH load in the PD-MCI group remained significantly increased. Mak et al. (45) also reported similar findings. However, a recent longitudinal study by Nicoletti et al. (34) found that baseline WMH load was not associated with the risk of PD-MCI, and severe baseline WMH was the strongest predictor of PDD. In our study, we compared the WMH load between the PD-NC and PD-MCI groups and found that the WMH load in the PD-MCI group was significantly higher than in the PD-NC group, suggesting that WMH may play an important role in early cognitive impairment in PD.

The evaluation of WMH can be classified into two methods: visual rating scales and volumetric assessments. Visual rating scales are commonly used as they are simple and quick; however, they have some subjective bias and ceiling effects, and their reliability and sensitivity are lower compared to volumetric measurement methods. Therefore, our study conducted subgroup analyses based on different WMH assessment methods, and found that even after excluding potential subjective bias in visual rating scales, significant associations between WMH and PD-MCI were still observed using the volumetric assessment.

As WMH load increases with age, and the prevalence of WMH positivity is significantly higher in Asians than in Caucasians (12), our study conducted subgroup analyses based on age-matching and ethnicity (Asian or Caucasian). We found that the impact of WMH load on early cognitive function in PD was not influenced by ethnicity or age; regardless of age-matching or ethnicity, the severity of WMH was significantly correlated with cognitive impairment in PD patients. Due to limitations in accessing research data, this study did not specifically explore the effects of WMH load on cognitive domains in PD.

Vesely et al. found that the impact of WMH load on cognitive function may vary in specific brain regions (55). In a cohort study, Kandiah et al. (41) reported that baseline PVH load significantly increased in the PD-MCI group compared to PD-NC, while there was no significant difference in DWMH load. Further univariate analysis revealed that PVH was significantly higher in PD-MCI than PD-NC; after adjusting for age and hippocampal volume in multivariate analysis, PVH only showed a certain trend, and DWMH was not a predictor of PD-MCI risk. Subsequently, Mak et al. (45) found that the overall WMH and PVH load were significantly higher in the PD-MCI group than in the PD-NC group, while there was no significant association between DWMH and PD-MCI. Interestingly, Li et al. (47) observed in a study of cognitive impairment in 120 PD patients that compared to PD-NC, DWMH load was significantly increased in PD-MCI, while there was no significant difference in PVH load between the two groups. Further multiple regression analysis demonstrated that the correlation between PD cognitive impairment and DWMH score was the highest. In our study, we found that both PVH and DWMH load were significantly associated with early cognitive function in PD patients, suggesting that both PVH and DWMH may affect early cognitive function in PD.

Two studies (42, 50) provided data on the correlation between lobar WMH load, basal ganglia WMH load, and PVH load with cognitive impairment in PD. Li et al. (50) reported that only frontal lobe WMH load showed a significant difference between the PD-MCI group and the PD-NC group, while Wang et al. (42) found no significant differences in WMH load in various brain regions between the PD-NC and PD-MCI groups. Due to limitations in accessing data, the effects of WMH load in different brain regions on cognitive impairment in PD were not explored based on each region’s WMH load. Future research on the effects of WMH in different brain regions on cognitive function in PD is expected to provide further insights into the impact of WMH in different brain areas on cognitive dysfunction in PD.

PD-MCI is the most crucial risk factor for PD progression to PDD (7). Compared to PD-MCI, PDD has a more significant impact on patients’ daily lives. Beyer et al. (51) first explored the impact of WMH on cognitive impairment in PD in 2006 and found that compared with the PDND group, the levels of DWMH load and PVH load were significantly higher in the PDD group. Multiple linear regression analysis revealed that DWMH was the only variable significantly correlated with MMSE scores. Subsequently, Joki et al. (36) observed that PVH load was significantly increased in PDD patients compared to PDND patients, while there was no significant difference in DWMH load between the two groups. In contrast, Slawek et al. (53) found in a study of 192 PD patients and 184 age-and gender-matched healthy controls that overall WMH load and DWMH load were related to cognitive impairment in PD patients, while no significant correlation was found with PVH. Further multivariate analysis showed that DWMH could predict PDD. Our study conducted a meta-analysis of studies on overall WMH load and PDD, and found that overall WMH load affects cognitive function in PDD patients. Further subgroup analysis revealed that both Asian and Caucasian WMH load were related to cognitive function in PDD, while there was no significant difference in WMH load between different age PDD and PDND groups, suggesting that age may confound the relationship between WMH and PDD. In future research, special attention should be paid to the impact of age on the relationship between WMH load and cognitive function in PDD.

## Limitations

This study has several limitations. First, unpublished data, case reports in abstract form, and non-English or Chinese literature were not included, which may have led to selection bias. Second, due to differences in analysis methods and designs among different studies, the comparability of data was limited, which may have had some impact on the research results. Third, due to data availability, this study did not analyze the relationship between WMH in different brain regions and cognitive domains in PD. Fourthly, this study did not take into account various sources of variation, including vascular risk factors, UPDRS scores, and the duration of PD. Therefore, more high-quality multicenter studies are still needed in the future to clarify the relationship between WMH and cognitive impairment in PD and provide more high-quality evidence-based medicine for clinical practice. This will allow for early and accurate identification and diagnosis of cognitive impairment in PD, reducing its harm and improving patient prognosis.

## Conclusion

WMH is associated with cognitive dysfunction in PD, and the severity of WMH may impact cognitive dysfunction in PD. Additionally, the early appearance of WMH may suggest the presence of MCI in PD.

## Data availability statement

The original contributions presented in the study are included in the article/supplementary material, further inquiries can be directed to the corresponding author.

## Author contributions

SZ proposed the concept of the review. WZ and BC performed data collection, analysis, and interpretation under the supervision of SZ and TZ. YC, YS, XF, ML, and YF provided reagents, materials, and analysis. WZ and BC wrote the manuscript. All authors approved the final version of the manuscript for submission.

## Funding

This work was supported by the Primary Health Development Research Center of Sichuan Province Program (Grants No. SWFZ21-Y-29) project for Nanchong City social science research “14th Five-Year Plan” project (Grants No. NC21B047).

## Conflict of interest

The authors declare that the research was conducted in the absence of any commercial or financial relationships that could be construed as a potential conflict of interest.

## Publisher’s note

All claims expressed in this article are solely those of the authors and do not necessarily represent those of their affiliated organizations, or those of the publisher, the editors and the reviewers. Any product that may be evaluated in this article, or claim that may be made by its manufacturer, is not guaranteed or endorsed by the publisher.

## Figures and Tables

**Table:** 

ARWMC	age-related white matter changes
BBB	blood–brain barrier
CHIPS score	the Cholinergic Pathways Hyperintensities Scale
CI	confidence intervals
CVR	cerebrovascular reactivity
DWMH	deep white matter hyperintensity
Erkinjunntti	the Erkinjunntti rating scales
Fazekas	the Fazekas visual rating scales
FLAIR	fluid-attenuated inversion recovery
H–Y stage	Hoehn and Yahr scale stage
MCI	Mild cognitive impairment
MD	mean differences
MMSE	Mini-Mental State Examination
MoCA	Montreal Cognitive Assessment
MOOSE	the Meta-Analysis of Observational Studies in Epidemiology guidelines
MRI	magnetic resonance imaging
Na	no available
NOS	Newcastle-Ottawa Scale
PD	Parkinson’s Disease
PDD	PD dementia
PD-MCI	Parkinson’s disease mild cognitive impairment
PD-NC	Parkinson’s disease with no cognitive impairment
PDND	non-demented PD
PVH	periventricular hyperintensity
Scheltens scale	the Scheltens visual rating scales.
SMD	standard mean difference
UKPDS	the use of British Parkinson’s Disease Society
UPDRS-III	Unified Parkinson’s Disease Rating Scale part III
WMH	White matter hyperintensity
